# CoVimmune COVID-19 Immunity Calculator: Web Application Development and Validation Study

**DOI:** 10.2196/59467

**Published:** 2025-04-22

**Authors:** Rebecca Slotkin, Tassos C Kyriakides, Vinni Yu, Xien Chen, Anupam Kundu, Shaili Gupta

**Affiliations:** 1 Department of Medicine, Yale School of Medicine, 950 Campbell Ave, Bldg 1, Floor 5, West Haven, CT, 06516, United States, 1 2038005320; 2 Cooperative Studies Program Coordinating Center, VA CT Healthcare System, West Haven, CT, 06516, United States; 3 Yale School of Public Health, Yale University, New Haven, CT, 06510, United States; 4 Department of Computer Science, Yale University, New Haven, CT, United States; 5 Yale Center for Analytical Sciences, Yale School of Public Health, New Haven, CT, United States; 6 Veterans Healthcare System of Connecticut, West Haven, CT, United States

**Keywords:** COVID-19, immunity, neutralizing antibody, immunoglobulin G, vaccine hesitancy, vaccine timing, patient-centered care, web application, vaccination, SARS-CoV-2

## Abstract

**Background:**

This study illustrates the development of a simple web-based application, which demonstrates the relationship between serum anti-SARS-CoV-2 S1/receptor-binding domain immunoglobulin G (IgG) and anti-SARS-CoV-2 neutralizing antibody (nAb) half-maximal inhibitory concentration (IC50) titers in a vaccinated US adult population and compares them to prior data on nAb titers at different time points after vaccination.

**Objective:**

The objective of this study is to create an easily accessible calculator that uses the results of commercially available anti-SARS-CoV-2 serum IgG to approximate the underlying ability to neutralize SARS-CoV-2.

**Methods:**

Our web-based application leveraged two previously published datasets. One dataset demonstrated a robust correlation between nAb and serum IgG. The other dataset measured nAb titers at specific time periods over a year-long interval following a messenger RNA vaccination primary series and booster vaccine dose. Clinical factors that were statistically significant on a forward linear regression model examining the prediction of nAb from serum IgG were incorporated in the application tool.

**Results:**

By combining the datasets described above, we developed a publicly available web-based application that allows users to enter a serum IgG value and determine their estimated nAb titer. The application contextualizes the estimated nAb titer with the theoretical distance from the corresponding vaccine-mediated antibody protection. Using the clinical variables that had a significant impact on how well IgG values predict nAb titers, this application allows for a patient-centered, nAb titer prediction.

**Conclusions:**

This application offers an example of how we might bring the advances made in scientific research on protective antibodies post-SARS-CoV-2 vaccination into the clinical sphere with practical tools.

## Introduction

High levels of anti-SARS-CoV-2 neutralizing antibodies (nAbs) have been shown to be protective against severe SARS-CoV-2 infection [1], but nAbs wane over time following vaccination or infection [2,3]. In addition, nAbs require significant funding and infrastructure to measure and are largely limited to research settings. Serum anti-SARS-CoV-2 immunoglobulin G (IgG) testing is available in commercial settings at relatively low cost. IgG testing has been sporadically ordered in primary care settings; although, there has been little to guide its clinical interpretation [4,5].

## Methods

### Ethical Considerations

Approval for the study from which both datasets were derived (SARS-CoV-2 Vaccine and You, protocol #1599488) was obtained from the Veterans Affairs Connecticut Healthcare System Institutional Review Board (IRB) in December 2020. Participants were eligible for inclusion if they were veterans or health care workers at the Veterans Affairs Connecticut Healthcare System and excluded if they were unable to provide informed consent. Written informed consent was obtained from willing participants prior to study participation and they were aware they could opt out at any time. The ethics approval covered secondary analysis as done for this paper without additional consent. Samples were donated by participants without any compensation and we are immensely grateful for this. All identifiable subject data was secured in encrypted folders accessible only to IRB-approved investigators. All data was de-identified before serological analysis.

### Source Data Sets

The first dataset was derived from a prospective cohort study which measured anti-SARS-COV-2 nAb half-maximal inhibitory concentration (IC50) titers against pseudotyped WA-1 (the original SARS-CoV-2 strain) at specific time periods over a year-long interval following the primary messenger RNA vaccination series and a subsequent booster vaccine dose (before dose 1, before dose 2, and after dose 2 at 1, 3, 6, and 12 months, with an additional collection 1 month after dose 3, which was approximately 10 months after dose 2). Clinical variables were collected by manual chart review as previously described. The nAb titers were found to fluctuate over time, peaking 1 month after the primary series, waning at 6 months, and rising again 1 month after the booster dose [2].

The second dataset leveraged the collected serum samples and measured IgG anti-S1/receptor-binding domain values in sera with predetermined nAb titers and clinical variables as previously described [6]. IgG and nAb IC50 titers had a robust linear correlation. Multimedia Appendix 1 illustrates this correlation for each clinical variable (COVID-19 infection status, estimated glomerular filtration rate (GFR), age, sex, history of malignancy, and race). Based on a forward stepwise linear regression, GFR (0‐30, 31‐59, and >60 mL/min/1.73 m²), and history of COVID-19 infection within the past 12 months (yes or no) were found to impact this correlation (*R*^2^=0.78) [6].

The model had undergone internal validation using repeated k-fold cross-validation. The model’s performance was evaluated using root mean squared error (RMSE), *R*^2^, and mean absolute error (MAE) across multiple versions of the model and different crossvalidation setups (ten 10-fold repeats, five 10-fold repeats, and three 5-fold repeats). For our model, which included IgG, COVID-19 infection status, and GFR, the RMSE was relatively low (around 0.27‐0.28) across validation setups, suggesting a small average error between the predicted and actual values. The *R*^2^ values were around 0.77 to 0.81 across validation setups, which indicated that the model explained about 77% to 81% of the variability in the data. The MAE values were around 0.2 for all validation setups, demonstrating a low average error magnitude (Multimedia Appendix 2).

### Application Development

#### Backend (Software)

The application used the model-view-controller pattern. We initially used RStudio 2023.12.1+402 running R 4.3.2 (IBM Corp) and an integrated development environment with a set of tools to complement R. The original R script had initially used its own web application framework (Shiny) to create a local web-based application, but this framework was not compatible with the rest of the tools used in the development process. The R script was converted to Python 3.9.12 (Python Software Foundation) for ease of web development. The main challenge when developing the project was converting the original R script into Python and creating the website using an HTML and cascading style sheets (CSS). Python was chosen over R for several reasons. First, Python’s versatility and ecosystem made integration with other tools like Flask, a popular web framework that simplifies the web application development process, and Docker, which simplifies the process of containerizing applications, much easier. Second, Python’s versatility and rich ecosystem of libraries and web development tools allowed us to keep the backend and front end the same language. Third, Python could handle a high volume of web requests and was better suited for scalable applications, which was a point of consideration. Last, Python was also the better choice for long-term maintainability, as it is more readable and easier to update. The host server emphasized Docker as the primary tool for deployment, making it the easiest tool to use for the development process. The full source code is available on request.

#### Front End (Web Interface)

The front end was converted from a Shiny package in RStudio to an HTML and CSS. The Python Flask 3.0.0 (Armin Ronacher) module was used to build the CoVimmune website. The website was launched using a YaleInformation Technology Support secured self-service sandbox environment called Spinup. The Waitress Web Server Gateway Interface was used to deploy our application and Docker 4.27.1 (Docker, Inc) was used to package, ship, and run our application.

## Results

**Figure 1.**
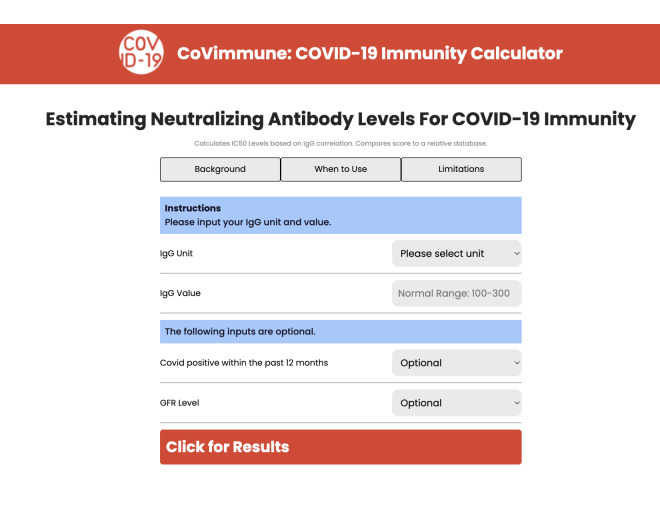
CoVimmune COVID-19 immunity calculator website interface for the application. GFR: glomerular filtration rate; IC50: half-maximal inhibitory concentration; IgG: immunoglobulin G.

**Figure 2.**
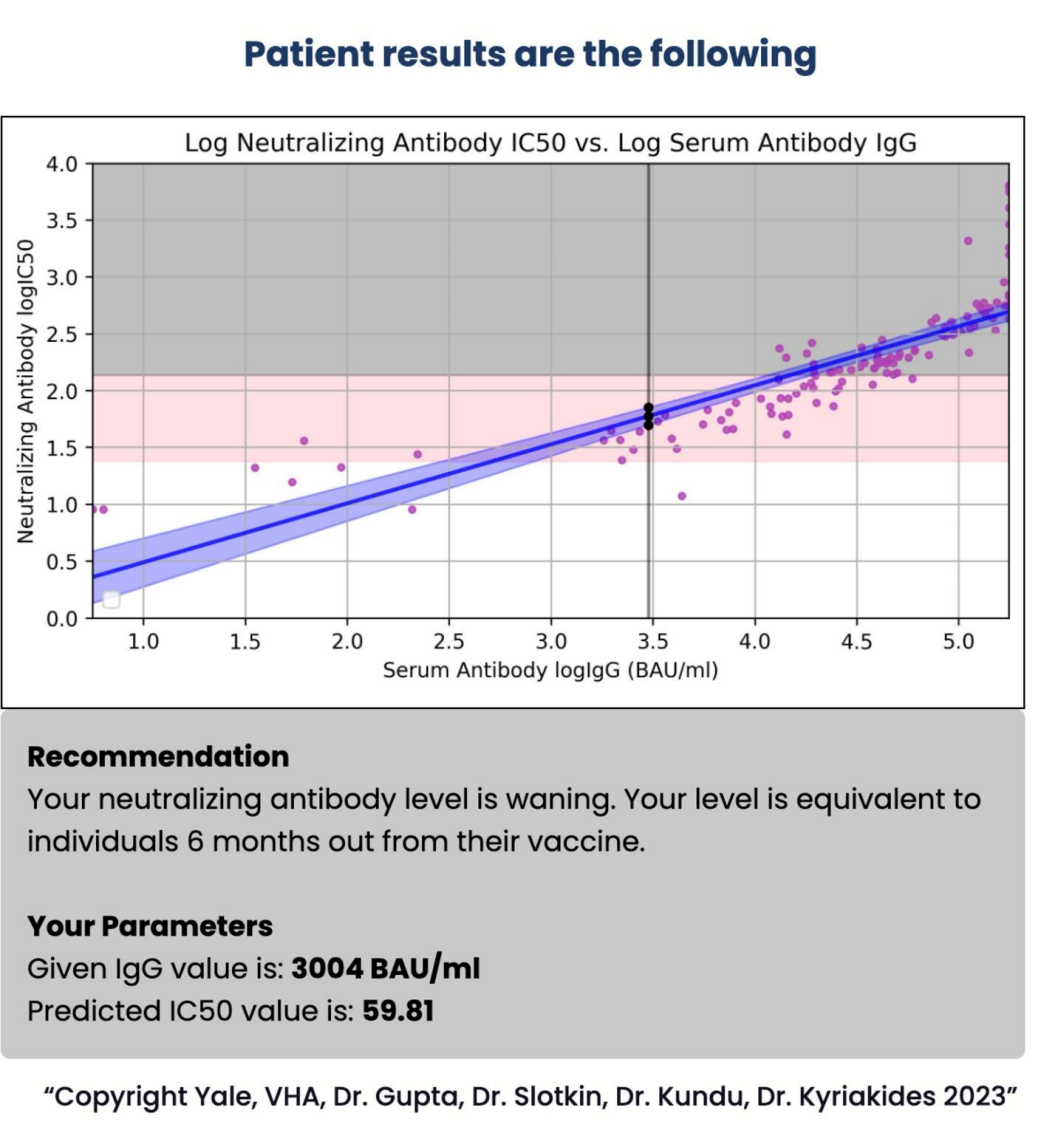
CoVimmune COVID-19 immunity calculator results with an example input. BAU: binding antibody unit; IC50: half-maximal inhibitory concentration; IgG: immunoglobulin G.

This web-based application was based on 127 sera samples in 100 unique participants aged 20‐93 years (mean 63.83, SD 15.63 years; 29% female; 67% White) from the second dataset. The user can input their IgG value, and the units can be from any of 4 commonly used platforms (Abbott-arbitrary U/ml, Siemens-U/ml, Roche-U/ml, and Euroimmune-relative U/ml) or the World Health Organization’s standardized IgG value in binding antibody U/mL. The user can choose to select their COVID-19 infection status and GFR category from drop down lists. The application then generates an estimated nAb titer, which is compared to individuals with matching renal function and COVID-19 status from the first dataset, to provide a contextualized range of nAb titers at different time points after vaccination (Figure 1 and Figure 2). The application also visualizes the results in the context of time from the last vaccination among individuals with similar values in the application dataset. The application is free and available on the internet [7].

## Discussion

The application illustrates the relationship between serum anti-SARS-CoV-2 IgG and anti-SARS-CoV-2 nAb IC50 titers in a vaccinated US adult population and allows for the adjustment of that relationship based on renal function and prior COVID-19 infection. It brings bench research into the clinical context and may provide a tool for physicians to personalize discussions about vaccine booster timing for patients where yearly vaccination may not be appropriate [8] or for those who are hesitant to get revaccinated.

This application is not intended to replace clinical judgment or offer conclusions about immune protection from COVID-19 or vaccine efficacy. The nAb titers are only one component of the immune response to infection. We cannot extrapolate cellular immunity from these data. This application is based on a small sample of patients and there were limited numbers of immunocompromised individuals within the sample, which may have limited generalizability to those individuals [9]. Ultimately, this application is a user-friendly example of how SARS-CoV-2 IgG nAb vaccine research, which has high barriers to replication and clinical application due to cost and infrastructure requirements, can be transformed into a practical tool.

## Supplementary material

10.2196/59467 Multimedia Appendix 1 Correlation between the log immunoglobulin G values and the log half-maximal inhibitory concentration titers by clinical variable.

10.2196/59467 Multimedia Appendix 2 Repeated k-fold cross validation results.

## Abbreviations

CSS: cascading style sheets
GFR: glomerular filtration rate
IC50: half-maximal inhibitory concentration
IgG: immunoglobulin G
MAE: mean absolute error
nAb: neutralizing antibody
RMSE: root mean squared error
