# Supplementary figures and images for: CoVimmune COVID-19 Immunity Calculator: Web Application Development and Validation Study
Source: JMIR Form Res. 2025 Apr 22;9:e59467. doi: 10.2196/59467 (PMC12040297; doi:10.2196/59467)

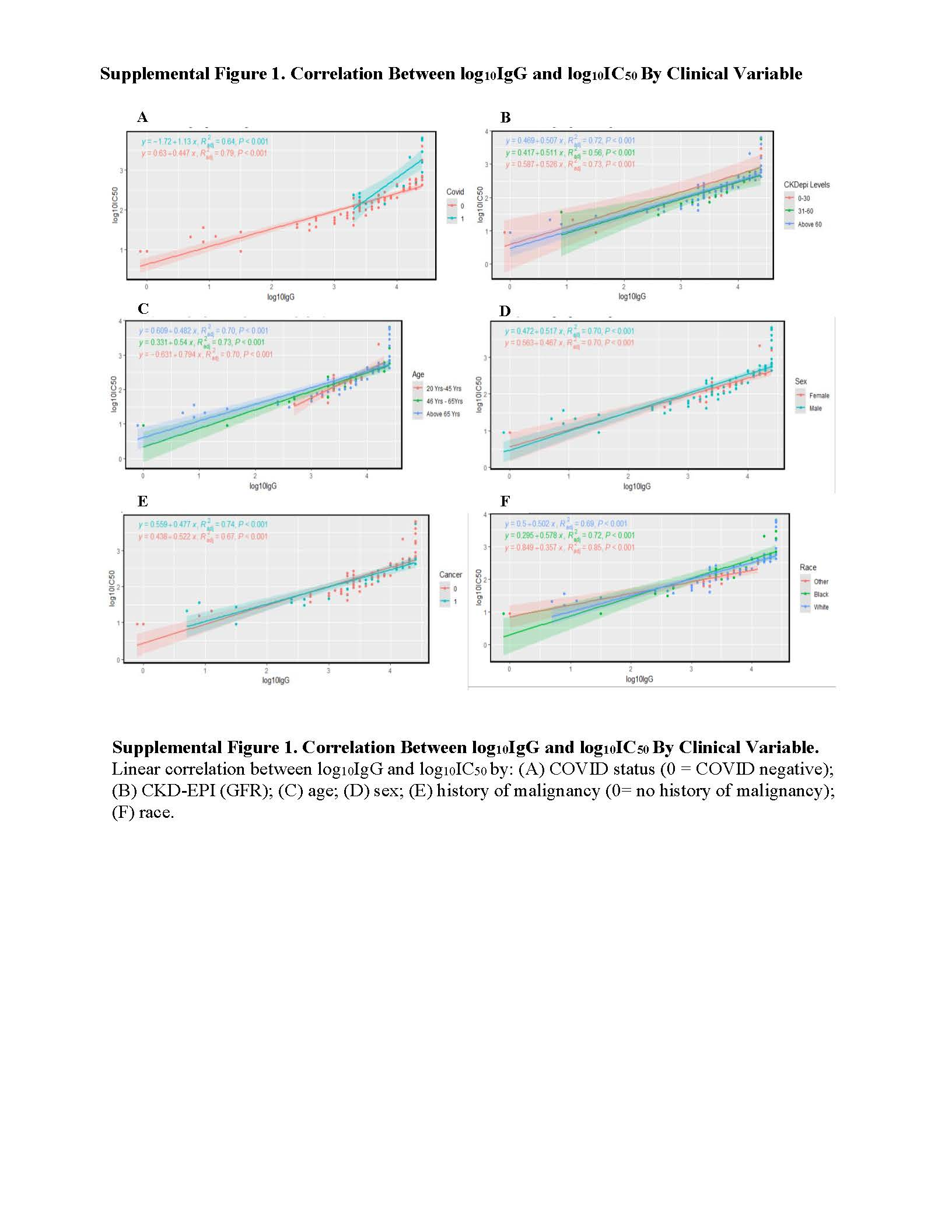

Supplement: Multimedia Appendix 1 [file formative-v9-e59467-s001.png]

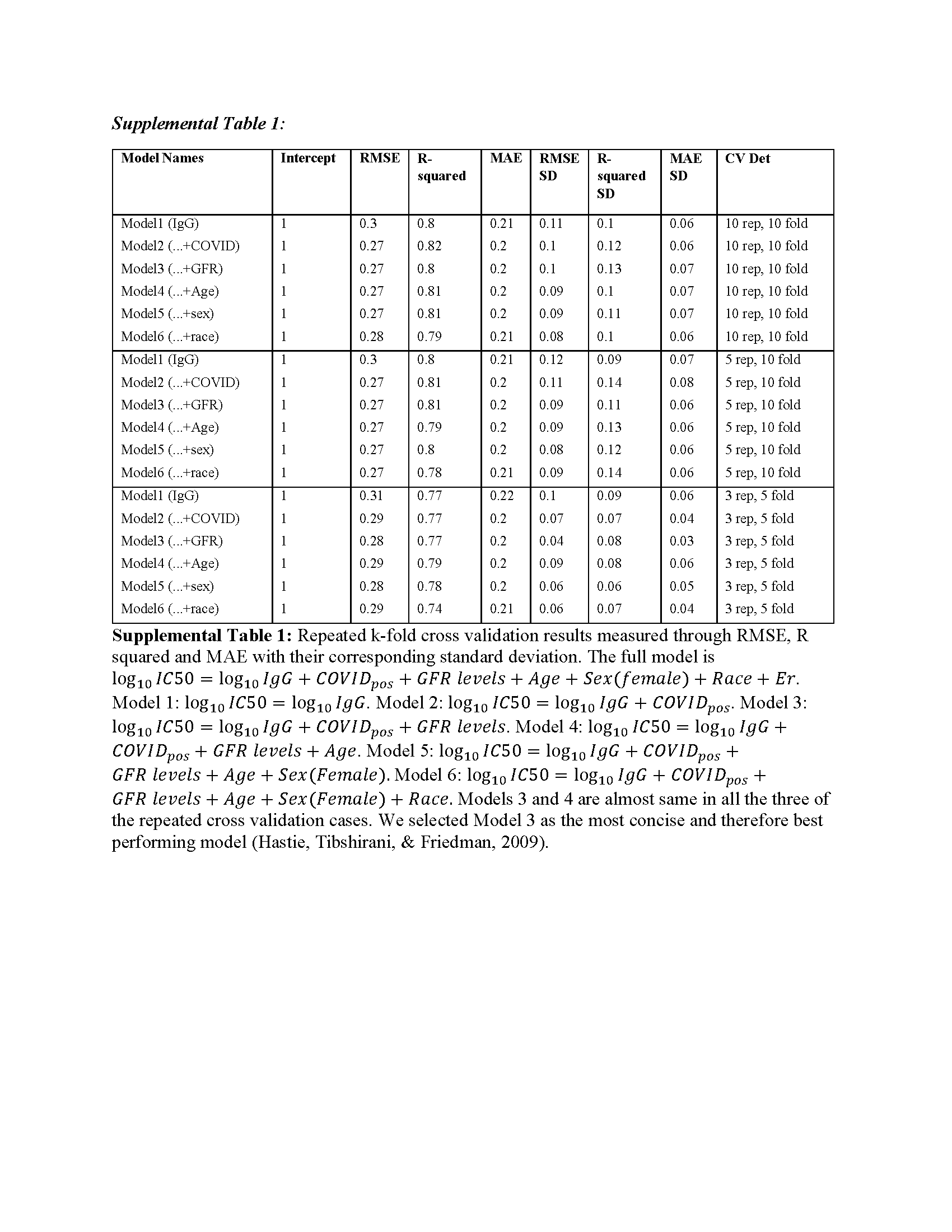

Supplement: Multimedia Appendix 2 [file formative-v9-e59467-s002.png]
